# A tau fragment links depressive-like behaviors and cognitive declines in Alzheimer’s disease mouse models through attenuating mitochondrial function

**DOI:** 10.3389/fnagi.2023.1293164

**Published:** 2023-12-06

**Authors:** Yamei Wang, Jianhao Wang, Hongyu Chen, Xiang Li, Ruifeng Xu, Feng Gao, Hang Yu, Fang Li, Dongdong Qin, Jiabei Wang, Yuke Shi, Yiyi Li, Songyan Liu, Xi Zhang, Shuai Ding, Yiqian Hu, Liqin Huang, Xin-Ya Gao, Zuneng Lu, Jin Luo, Zhi-Hao Wang

**Affiliations:** ^1^Department of Neurology, Renmin Hospital of Wuhan University, Wuhan, China; ^2^Center for Neurodegenerative Disease Research, Renmin Hospital of Wuhan University, Wuhan, China; ^3^Union Hospital, Tongji Medical College, Huazhong University of Science and Technology, Wuhan, China; ^4^Department of Neurology, Henan Provincial People's Hospital, Zhengzhou, China; ^5^Laboratory of Neurology, Henan Provincial People’s Hospital, Zhengzhou, China; ^6^Center for Reproductive Medicine, Renmin Hospital of Wuhan University, Wuhan, China

**Keywords:** tau, depression, Alzheimer’s disease, mitochondria, PPAR-δ

## Abstract

**Introduction:**

Alzheimer’s disease (AD) is the most prevalent neurodegenerative disease characterized by extracellular senile plaques including amyloid-β peptides and intracellular neurofibrillary tangles consisting of abnormal Tau. Depression is one of the most common neuropsychiatric symptoms in AD, and clinical evidence demonstrates that depressive symptoms accelerate the cognitive deficit of AD patients. However, the underlying molecular mechanisms of depressive symptoms present in the process of AD remain unclear.

**Methods:**

Depressive-like behaviors and cognitive decline in hTau mice were induced by chronic restraint stress (CRS). Computational prediction and molecular experiments supported that an asparagine endopeptidase (AEP)-derived Tau fragment, Tau N368 interacts with peroxisome proliferator-activated receptor delta (PPAR-δ). Further behavioral studies investigated the role of Tau N368-PPAR-δ interaction in depressive-like behaviors and cognitive declines of AD models exposed to CRS.

**Results:**

We found that mitochondrial dysfunction was positively associated with depressive-like behaviors and cognitive deficits in hTau mice. Chronic stress increased Tau N368 and promoted the interaction of Tau N368 with PPAR-δ, repressing PPAR-δ–mediated transactivation in the hippocampus of mice. Then we predicted and identified the binding sites of PPAR-δ. Finally, inhibition of AEP, clearance of Tau N368 and pharmacological activation of PPAR-δ effectively alleviated CRS-induced depressive-like behaviors and cognitive decline in mice.

**Conclusion:**

These results demonstrate that Tau N368 in the hippocampus impairs mitochondrial function by suppressing PPAR-δ, facilitating the occurrence of depressive-like behaviors and cognitive decline. Therefore, our findings may provide new mechanistic insight in the pathophysiology of depression-like phenotype in mouse models of Alzheimer’s disease.

## Introduction

Alzheimer’s disease (AD), the most prevalent neurodegenerative disease, is primarily characterized by deterioration of cognitive functions and commonly accompanied with neuropsychiatric symptoms. The two main pathological features in AD brain are extracellular senile plaques composed of the amyloid-β peptides and intracellular neurofibrillary tangles consisting of abnormal Tau (Bloom, 2014). Depressive symptoms cause a serious public health problem all over the world, with a pronounced impact on those patients with neurodegenerative disease, especially those with AD (Reus et al., 2016). Considerable evidences have shown that depression is related to increased risk for developing AD later in life (Herbert and Lucassen, 2016). However, the molecular mechanisms underlying the development of depression and depressive symptoms in AD are still unclear.

Both researches on postmortem interval and pre-clinical murine models have shown that mitochondrial dysfunction is associated with AD pathophysiology (Lynn et al., 2010; Martins et al., 2017; Mancini et al., 2021). The major mitochondrial dysfunction in AD is apparent from a decrease in neuronal ATP levels and the overproduction of reactive oxygen species (ROS) (Alqahtani et al., 2023). Recent studies have shown that rescue of mitophagy deficits by promoting mitophagosomes fusion can attenuate Aβ accumulation, relieving cognitive decline in 5 × FAD mice (Chen et al., 2021). Additionally, abnormal energy metabolism caused by impaired mitochondria is related to depressive symptoms as lower energy production of cortical neurons from depressed patients (Ernst et al., 2017). A recent study has demonstrated that glucocorticoids, released in response to chronic stress, can promote mitochondrial damage and Tau phosphorylation and oligomerization, leading to depressive-like behaviors in mice (Du et al., 2023). Strikingly, exogenous ATP administration or stimulating endogenous ATP release can rescue depressive-like behaviors in mice (Cao et al., 2013). Based on the above findings, we speculated that mitochondrial dysfunction could participate in the onset and progress of depressive symptoms in AD mice.

Tau is a microtubule-associated protein and its main function is to keep microtubules stable and promote microtubules assembly (Tapia-Rojas et al., 2019). Under pathological conditions, abnormal phosphorylation and cleavage of Tau can lead to the pathogenesis of AD (Tapia-Rojas et al., 2019). It has been well established that abnormal phosphorylation of Tau accelerates the onset of AD, but abnormal truncation of Tau still needs to be fully studied. Recent studies reported that mammalian asparagine endopeptidase (AEP, gene name *LGMN*), a proteolytic enzyme, can cleave Tau at N255 and N368 residues and produce toxic truncated fragments which would promote Tau aggregation, leading to the exacerbation of AD-like pathologies in mice (Zhang et al., 2014). In addition, accumulating clinical studies have indicated Tau pathology is modestly associated with depressive symptoms, but the underlying mechanisms remain elusive (Moriguchi et al., 2021). Moreover, a N-terminal Tau fragment has been reported to target neuronal mitochondria at AD synapses (Amadoro et al., 2015). Hence, we hypothesized that a truncated Tau N368 (Tau 1–368) fragment derived from AEP triggers mitochondrial dysfunctions, promoting depressive-like phenotype in AD mouse models.

In this study, we performed a 14-day procedure of chronic restraint stress (CRS) to induce depressive-like behaviors in mice. The results showed that CRS not only induced depressive-like behaviors, but also impaired cognitive function in hTau mice, which is a mouse model lacking murine Tau but expressing human Tau. Further experiments demonstrated that hippocampal Tau N368 fragment, elevated by CRS, directly binds to and inhibits peroxisome proliferator-activated receptor δ (PPAR-δ), a transcription factor abundant in the brain that promotes mitochondrial biogenesis, leading to depressive-like behaviors and cognitive impairments in AD mouse models.

## Materials and methods

Antibodies, chemicals, and virus resources: Anti-PPAR-δ antibody, Proteintech, 60193-1; Anti-Tau N368 antibody, Keqiang Ye lab; Anti-FLAG antibody, Abways, AB0008; Anti-GST antibody, Proteintech, 66001–2; Anti-GFP antibody (B2), Santa Cruz, sc-9996; Anti-α-Tubulin antibody, Sigma-Aldrich, T6074. 4′,6-diamidino-2-phenylindole (DAPI), Sigma-Aldrich, D9542; AEP inhibitor-compound 11 was purchased from J&K Scientific Ltd. (Beijing, China). GW0742 (HY-13928), was purchased from MedChem Express; HEK 293, ATCC, CRL-1573. AAV-GFP-Tau N368, AAV-PPAR-δ-FLAG, and AAV-PPAR-δ mKNK-FLAG were purchased by Brain Case Technology (Shenzhen). It is worth mentioning that GFP and FLAG were fused to the target protein in the viruses we used.

### Animals

Wild-type (WT) C57BL/6 J mice, hTau mice, and 3xTg mice were purchased from the Jackson Laboratory (stock #000664, #005491, and #034830 respectively). The hTau mice lack endogenous microtubule-associated Tau (MAPT) gene, and express human MAPT gene. All mice were housed and kept under specific pathogen-free (SPF) conditions with a 12 h/12 h light/dark cycle at a constant temperature (22 ± 2°C). The supply of food and water is adequate to all mice, unless otherwise indicated. Mice were randomly assigned to each group by using a random number table. The number of mice used in the experiments was determined by Power and Precision (Biostat). Before behavioral tests, mice were randomly taken from each group. All animal experimental protocols were approved by the Laboratory Animal Welfare Ethical Committee of Renmin Hospital of Wuhan University (No. WDRM 20230706D). We performed the animal care and handling according to the NIH animal care guidelines and Wuhan University guidelines. All procedures related to mice followed the ethical standards of the Renmin Hospital of Wuhan University Institutional Animal Care and Use Committee.

### Study design

At first, we applied acute restraint stress (ARS) and CRS to WT and hTau mice, followed by a series of behavioral tests including open-field test (OFT), sucrose preference test (SPT), forced swim test (FST), novel object recognition test (NOR) and contextual fear conditioning and cued fear conditioning. According to the results of behavioral tests, we performed correlation analysis of depressive-like behaviors and cognitive decline. Next, to understand the role of mitochondria in depressive-like behaviors and cognitive decline, we detected the mitochondrial function and the interaction of Tau N368 and PPAR-δ. Further, to explain the molecular mechanism of mitochondrial dysfunction, we screened the binding region in which Tau N368 binds to PPAR-δ by immunoprecipitation and GST-pulldown and further identified the binding sites of Tau N368 and PPAR-δ. Then, point mutation of binding sites in PPAR-δ disrupted the binding and relieved the behavioral disorders. Finally, we used AEP inhibitor, anti-Tau N368 antibody and PPAR-δ agonist to confirm our hypothesis in hTau and 3xTg mice. All the animal experiments were performed by a blind investigator.

### Antibody administration in mice

The hTau and 3xTg mice (15 mice per group) were injected intraperitoneally with 2 mg/kg twice a week during 14 days of CRS procedure. Each mouse was randomly received Tau N368 antibody as experimental group or IgG in saline as control group. After CRS with administration, we performed a series of behavioral tests on the mice.

### Preparation and administration of test agents

Compound 11 was first dissolved in dimethyl sulfoxide as the stock solution and then diluted in 0.9% NaCl solution containing gum arabic for systemic treatment. The mice received randomly vehicle or compound 11 at a dose of 10 mg/kg once daily for 60 days through gavage treatment. On day 22, mice were exposed to CRS and subsequent behavioral and molecular experiments.

GW0742 was dissolved in 1% dimethyl sulfoxide, 1% Tween-80 and 98% phosphate-buffered saline solution at 50 mM concentration. After CRS, mice were injected intraperitoneally with vehicle or GW0742 once daily at a dose of 5 mg/kg for a week, then exposed to behavioral and molecular experiments.

### Stress procedures

This procedure was conducted with slight modifications based on a previously described method (Seo et al., 2012). The mice were individually housed to adapt the environment for a week, then randomly were divided into the control group, ARS group and CRS group. The mice received restraint stress (RS) were confined into the crylic cylindrical flat-bottom head-first restrainer. ARS mice only experienced the restraint stress once for 2 h, while CRS mice experienced the restraint stress for 2 h per day for 14 days, or 21 days (RS21). The control mice remained in their home cages without any stress.

### Behavioral tests

All test mice were habituated in a behavioral testing room for 3 days, accompanied with daily handling for 1 min. The order of the behavioral tests is to start with the least stressful test and leave the most stressful of all for last. To avoid mutual influence between different behavioral experiments, mice were given at least 1 day to recover from the last behavioral test.

### Tail suspension test

The mice were suspended individually by adhering their tails with a 15 cm-long tape, and their nose were positioned 20–25 cm above the floor. The definition of immobility is the mice were hung passively without any initiated movements. The immobile time was recorded during the total time of 6 min. The ratio of immobile time to total time reflects the degree of depressive-like behaviors in mice.

### Forced swim test

The mice were individually placed into a transparent glass cylinder (height 35 cm and diameter 15 cm) filled with 30 cm-depth of warm water (25 ± 1°C). We renewed the water after each test. The definition of immobility is the mice were floating without any initiated movements, expect those motions essential to keep balance in the water. All mice were forced to swim for 6 min, and their immobility time was recorded during the final 5 min of the test. The ratio of immobile time to the recorded time reflects the degree of depressive-like behaviors in mice.

### Sucrose preference test

We performed a 6-day sucrose preference protocol to evaluate the depressive-like behaviors in mice. The mice were housed individually, provided with two bottles of normal water for 2 days and two bottles of 2% sucrose solution for the following 2 days. After that, mice experienced deprivation of water and food for 1 day and then received access to two bottles, the one filled with a 2% sucrose solution and the other filled with normal water for 24 h in the dark. The positions of the bottles were switched after 12 h. Each bottle was weighed twice, initial weight and final weight, and the difference between the two weights was the consumption of normal water or sucrose solution. The definition of sucrose preference, the amount of sucrose solution consumed was divided by the sum of both water and sucrose solution ingested during the last day and then expressed as a percentage. The less than 65% sucrose preference reflects depressive-like behaviors in mice (Strekalova et al., 2004; Scheggi et al., 2018).

### Open-field test

Animals were placed in a rectangular chamber (50 × 50 × 50 cm^3^) made of opaque white plastic. The movement distance of mice was monitored and recorded using ANY-maze software (Stoelting CO, United States) for 6 min. The movement distance of mice reflects the locomotor activity.

### Novel object recognition test

The apparatus for novel object recognition test is the same as the open-field apparatus. The novel object recognition test has two parts, NOR familiar-pretest and NOR novel-test. On the first day of the test, two identical objects (familiar objects) were presented to the mice. These objects were located at the left and right corners of the area, and the mice were given 5 min to freely explore the two objects. The next day, one of the familiar objects was replaced with a new object (novel object), and again allowed the mice to explore for 5 min. The test on the first day is NOR familiar-pretest and the test on the next day is NOR novel-test. The definition of exploration is touching the object (tail-touching and climbing excluded), sniffing the object, or facing the object (distance <2 cm). We used ANY-maze software to record the duration of time spent examining each object, whether it was familiar or novel. The discrimination index is calculated as the ratio of time spent exploring the novel object versus the total time spent exploring both the two objects or both the familiar and novel objects. The discrimination index of mice reflects the spatial recognition memory.

### Fear conditioning test

The apparatus of fear conditioning test is a box measuring 17 × 17 × 35 cm^3^, equipped with a ceiling mounted video tracking camera, a light, a speaker, a soundproof door, and an electric shock floor. In the contextual fear conditioning test, on the first day, the mice were put into the apparatus for 3 min, followed by 2 s, 0.4 mA foot shock three times with an interval of 1 min. The next day, the mice were again put into the same conditioning chamber for 3 min and the freezing time was record. In the cued fear conditioning test, the protocol of the first day is almost consist with the contextual fear conditioning test apart from the foot shock paired with tone stimulate. The next day of the cued fear conditioning test, the mice were put into a novel chamber which is different from the first chamber and given three tones stimulate with an interval of 1 min, and the freezing time was record. The definition of freezing is “absence of movement except for respiration.” The freezing percentage is calculated as the ratio of freezing time versus the total time. The freezing percentage of mice reflects the ability to learn and remember an association between environmental cues and aversive experiences.

### Immunoprecipitation

The hippocampal tissues or cells tissues were ground and then lysed on ice for 30 min, using lysis buffer containing of protease inhibitors and phosphatase inhibitors. The samples were centrifuged at maximum speed for 15 min at 4°C, and the protein supernatants were collected. The protein concentration of supernatants was homogenized in lysis buffer, then the samples were incubated with the primary antibodies overnight at 4°C, followed by adding protein A/G agarose for 4 h and rotating at 4°C. Finally, the samples were centrifuged at 2500 rpm for 5 min at 4°C, then the supernatants were removed and the protein A/G agarose was added with SDS loading buffer and boiled at 95°C for 10 min. The collective samples were analyzed by western blotting.

### GST-pulldown

The protein supernatants collected from brain tissues or cell tissues were incubated with the Glutathione Sepharose™ 4B beads (17,075,601, Cytiva) overnight and rotating at 4°C. After 24 h, the GST beads were deposited at the bottom of the tube after centrifuged at 500 G for 5 min, and the GST-tagged protein bind to beads and were congregated. SDS loading buffer was added into samples and boiled 10 min at 95°C for western blotting.

### Western blotting

The bilateral hippocampal tissues and the cell tissues were ground and then lysed on ice for 30 min, using 80 μL of lysis buffer containing of protease inhibitors and phosphatase inhibitors. The protein samples were then centrifuged at maximum speed for 15 min at 4°C, and the supernatant was collected. Protein quantification was carried out using the Thermo BCA Protein Assay Kit (Cat#23227). Then, the protein supernatants were boiled in SDS loading buffer for 10 min at 95°C, separated by 10% SDS-PAGE, and transferred onto nitrocellulose membranes. The membranes were blocked with 5% skim milk for 1 h at room temperature and incubated overnight at 4°C with the appropriate primary antibodies. After washed 4–6 times with Tris-buffered saline containing 0.1% Tween-20 (TBS-T), the membranes were incubated for 1 h at room temperature with horseradish peroxidase (HRP)-conjugated anti-mouse secondary antibodies (1:5000; BL001A, Biosharp Life Sciences, China) and anti-rabbit secondary antibodies (1:5000; BL003A, Biosharp Life Sciences, China). The membranes were washed again 6–8 times with TBS-T, then immersed in enhanced chemiluminescence reagents and imaging using the ChemiDoc™ Touch Imaging System. The bands were analyzed using the ImageJ software, with tubulin serving as the loading control. Each experiment was repeated and analyzed at least 3 times.

### Protein–protein docking prediction

The 3D protein structure of PPAR-δ has been deposited in the Protein Data Bank, www.pdb.org (PDB ID codes, 3TKM). The 3D protein structure of Tau FL (1–441) has been deposited in PDB-Dev database, www.pdb-dev.wwpdb.org (PDBDEV_00000033). The 3D protein structure of Tau N368 was derived from the Tau FL (1–441) structure with the exclusion of the domain (369–441). Computational docking of Tau N368 and PPAR-δ was performed to predict the complex structure between both proteins, using HDOCK webserver (Yan et al., 2020). This software employs a fast Fourier transform–based search strategy to model various potential binding modes between proteins. Subsequently, each binding mode undergoes evaluation using the ITScorePP scoring function. We retained the top three models and integrated the docking outcomes with our experimental data to make the final model selection.

### Luciferase assay

Primary neurons were seeded in 6-well plates. To evaluate transcriptional activity of PPARδ, a 3 × PPRE-luciferase reporter construct (addgene, #1015) with Renilla luciferase vector (addgene, #118016) were co-transfected into primary neurons. Cells were harvested using a passive lysis buffer at 24 h after transfection and analyzed using a Dual-Luciferase Reporter Assay System according to the manufacturer’s protocol (Promega) on a microplate reader. Relative light units of PPAR-δ WT or PPAR-δ mKNK luciferase were normalized to Renilla luciferase light units to control for transfection efficiency. The experiments were performed in three times.

### Relative mitochondrial copy number

The relative mitochondrial DNA (mtDNA) copy number means the ratio of mtDNA versus nuclear DNA, which measured by qPCR. We used QIAamp DNA Mini Kit (51,304, Qiagen) to extract total DNA. The primers of mitochondrial DNA: forward: 5’-GCCAGCCTGACCCATAGCCATAAT-3′ and reverse: 5’-GCCGGCTGCGTATTCT-ACGTTA-3′. The primers of nuclear DNA: forward: 5’-TTGAGACTGTGATT-GGCAATGCCT-3′ and reverse: 5’-CCAGAAATGCTGGGCGTACT-3′. PCRs were performed in triplicate. Real-time PCR probes were bought from TaqMan® (Thermo Fisher Scientific).

### Mitochondrial membrane potential assay

We used the JC-1 assay kit (Beyotime Biotech, China) to detect mitochondria membrane potential (MMP) based on the manufacturer’s instruction. For brain tissues, the hippocampus needs to be distinguished quickly and mitochondria was isolated by mitochondria isolation kit (Beyotime Biotech, China) according to the instructions. For primary neurons, JC-1 was added to monitor Δψm in a 5% CO_2_ incubator at 37°C for 30 min, then rinsed with PBS. The fluorescence signals of JC-1 monomer were monitored at 490 nm excitation (ex)/530 nm emission (em), and the fluorescence signals of J-aggregates of JC-1 were monitored at 525 nm excitation (ex)/590 nm emission (em). The addition of 3-chlorophenylhydrazone (CCCP) collapsed the proton gradient and served as a positive control for depolarization.

### Quantification of ATP

We used an ATP Assay Kit (S0026, Beyotime) to determine ATP levels in hippocampal CA1 tissues. Briefly, ATP was isolated from the hippocampus and lysed with ATP lysis buffer. After protein isolation and measurement, the reaction mixture containing protein samples or standard (100 μL) with ATP assay solution (100 μL) was incubated for 3–5 min at room temperature. Fluorescence was measured using a microplate reader. The ATP levels were calculated based on the standard curve and expressed in nmol/mg protein.

### Immunofluorescence staining

Free-floating 20 μm brain sections were used for immunofluorescence staining. The brain sections were washed three times with PBS, blocked with 3% bovine serum albumin (BSA, w/v) and 0.3% Triton X-100 (v/v) for 1 h at room temperature, and then incubated with primary antibodies at 4°C overnight (anti-Tau N368, 1:500). On the second day, after washed with PBS three times, the slices were incubated at 37°C with a mixture of labeled secondary antibodies (Alexa Fluor® 488) for 1 h. After washing with PBS three times, DAPI (1:1000) was added for nucleus staining for 15 min. Images were captured using a Leica Confocal Imaging System.

### Quantification and statistical analysis

The figures and figure legends show the sample size, the number of mice for each group used in this study. All data are analyzed by using GraphPad Prism Software and are presented as mean ± standard error of the mean (SEM) from three or more independent experiments. Each figure legend presents all the statistical procedures, containing the statistical tests and the number of mice or samples used. We use an unpaired *t*-test with Welch’s correctio to compare group pairs, one-way ANOVA and Bonferroni’s multiple comparison test to multiple-group comparisons. Statistical significance was indicated by *p* < 0.05.

### Data availability

The data that support the findings of this study are available on request from the corresponding author.

## Results

### Chronic restraint stress induces depressive-like behaviors and cognitive deficits in the hTau mice

**Figure 1:**
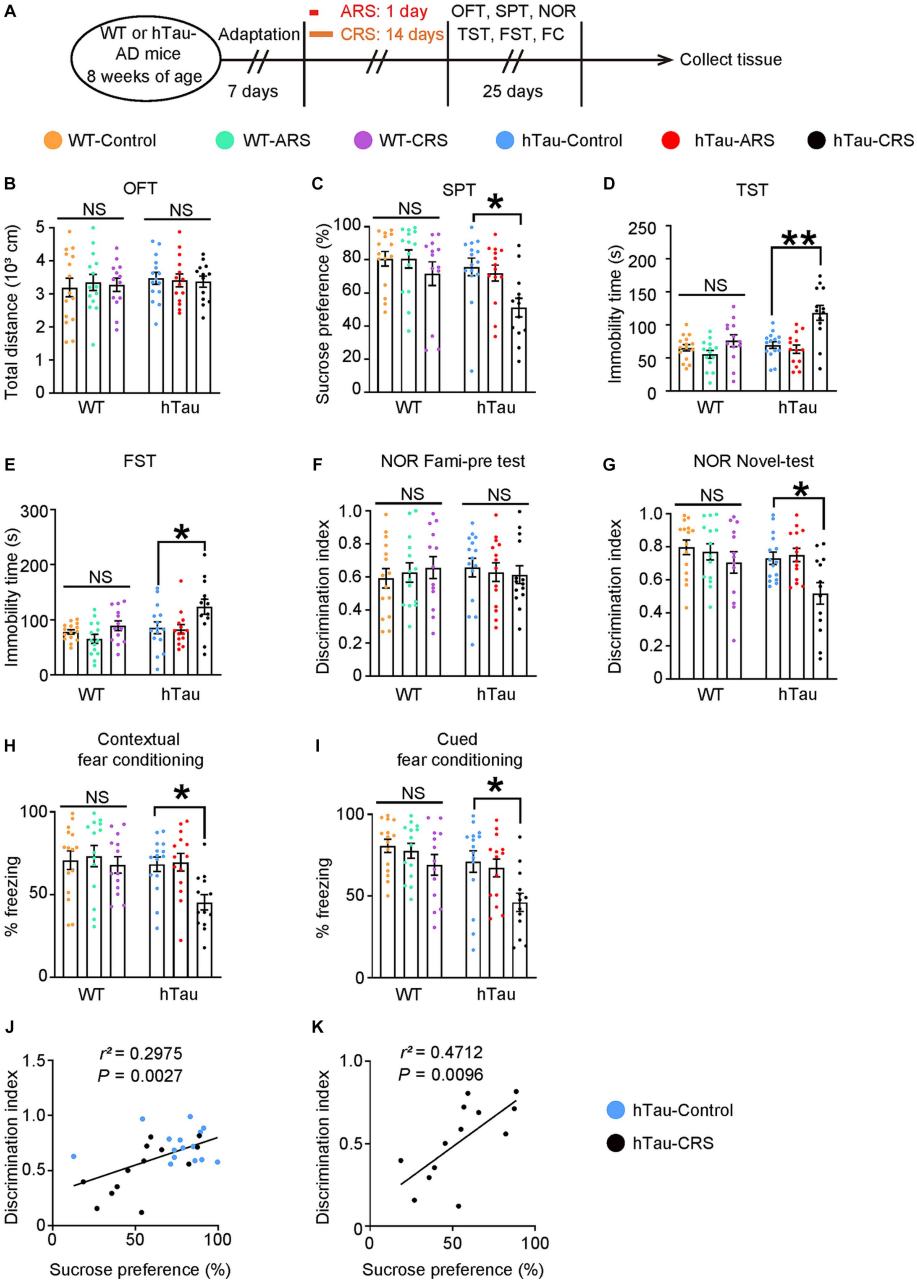
Chronic stress induces depressive-like behaviors and cognitive deficits in the hTau mice. **(A)** Schematic of ARS, CRS or control course and behavioral tests plan. ARS, acute restraint stress; CRS, chronic restraint stress; OFT, open-field test; SPT, sucrose preference test; NOR, novel object recognition test; TST, tail suspension test; FST, forced swim test; FC, fear conditioning. **(B)** Open-field test, **(C)** sucrose preference test, **(D)** tail suspension test, **(E)** forced swim test, **(F,G)** novel object recognition test, **(H)** contextual fear conditioning, **(I)** cued fear conditioning tests results. Experiments were performed on 8-week-old WT or hTau-AD mice received ARS, CRS or control produces. Results are presented as the mean ± SEM (*n* = 15 mice for control group, *n* = 14 mice for ARS group, *n* = 13 mice for CRS group; NS, not significant; *p < 0.05; **p < 0.01; one-way ANOVA and Bonferroni’s multiple comparison test). **(J)** Correlation between the discrimination index and the sucrose preference in hTau-Control and CRS groups. **(K)** Correlation between the discrimination index and the sucrose preference in hTau-CRS group. Quantitative analysis of the correlation between the discrimination index and the sucrose preference. The Spearman correlation coefficient *r*^2^ and *p* value are shown. Blue dots, hTau-Control. Black dots, hTau-CRS.

To explore the molecular mechanisms underlying the link between AD and depression, we applied RS procedures in WT mice and hTau-AD mice at the age of 8 weeks. After being immobilized in the restrainer (2 h/day) for 1 day (ARS procedure) or consecutively for 14 days (CRS procedure), mice were exposed to behavioral tests to evaluate the cognitive function and depressive-like behaviors (Figure 1A). All groups of mice displayed similar spontaneous locomotor activity after stress procedures as the total distance traveled in the OFT was not affected (Figure 1B). Notably, compared to the control group, hTau mice exposed to CRS but not ARS developed depressive-like behaviors, as evidenced by lower sucrose preference in the SPT and elevated immobility time in the TST and FST, whereas these alterations were not observed in WT mice (Figures 1C–E). Considering mice with depressive-like behaviors exhibit elevated freezing time in water, which makes Morris water maze test unreliable, we conducted the NOR and FC tests to evaluate the cognitive deficit in mice. Importantly, those above mice showed depressive-like behaviors also displayed significant memory deficiency, as supported by lower discrimination index in the NOR and decreased freezing percentage in the FCT, and these alterations were similarly absent in all WT mice (Figures 1F–I). Furthermore, as shown in Figures 1J,K, the discrimination index was positively correlated with sucrose preference in hTau mice (*r^2^* = 0.2975), particularly in the hTau-CRS mice (*r^2^* = 0.4712). These results indicate that CRS can simultaneously induce both depressive-like behaviors and cognitive dysfunctions in hTau mice.

### Mitochondrial dysfunction is involved in CRS-induced depressive-like behaviors and cognitive decline in hTau mice

**Figure 2:**
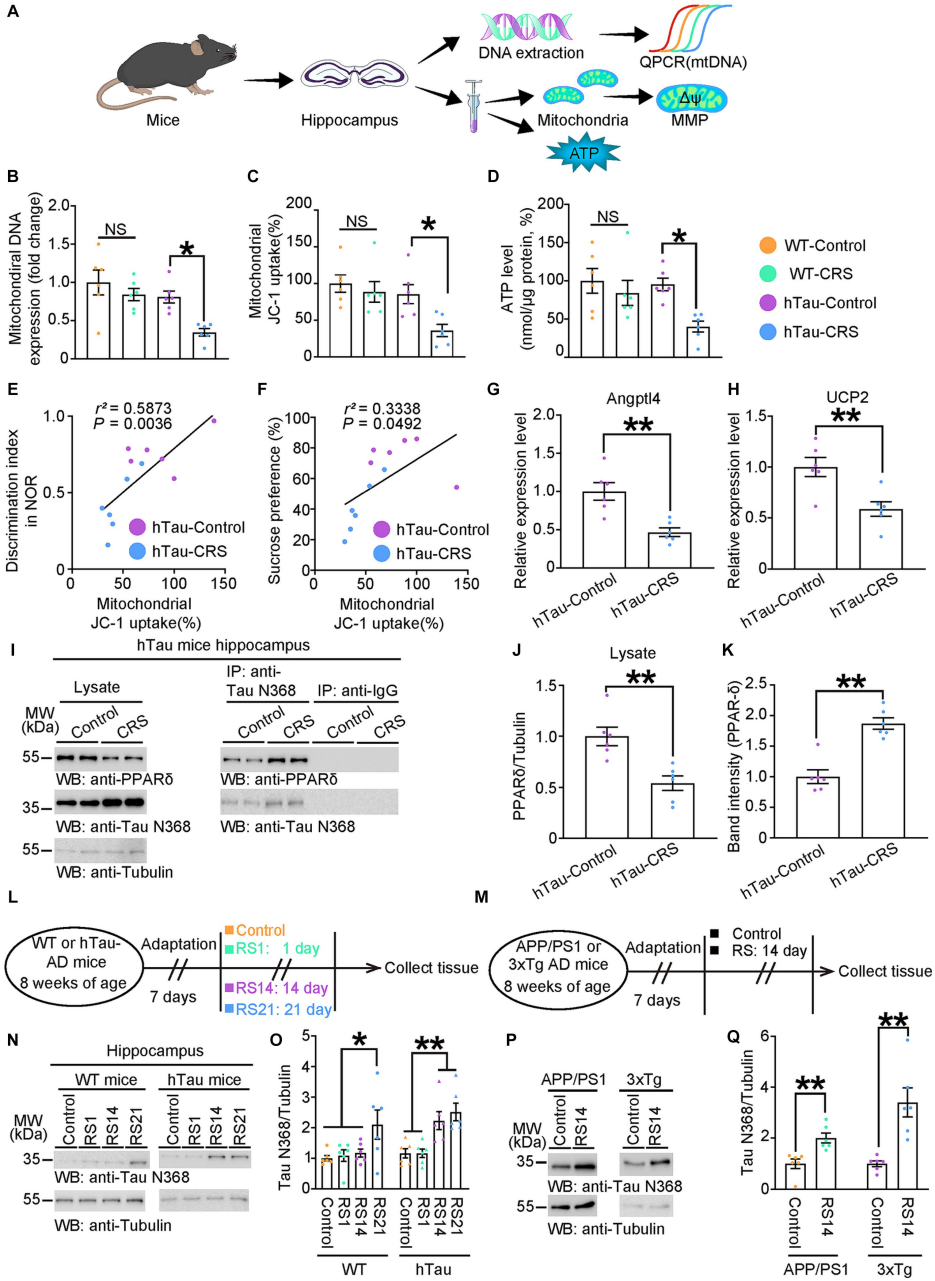
Mitochondrial dysfunction involves in CRS-induced depressive-like behaviors and cognitive decline in hTau mice. **(A)** Schematic experiment procedures of mtDNA, MMP, and ATP tests in the control and CRS groups. **(B–D)** Measurement of mitochondrial DNA copy number **(B)**, MMP **(C)**, and ATP levels **(D)** in the hippocampus of control and CRS mice. Data represent mean ± SEM (*n* = 6 for each group; NS, not significant; *p < 0.05; one-way ANOVA and Bonferroni’s multiple comparison test). **(E)** Correlation between the discrimination index and the mitochondrial JC-1 uptake in hTau-Control and hTau-CRS groups. **(F)** Correlation between the sucrose preference and the mitochondrial JC-1 uptake in hTau-Control and hTau-CRS groups. Quantitative analysis of the correlation between the discrimination index or the sucrose preference and the mitochondrial JC-1 uptake. The Spearman correlation coefficient *r*^2^ and *p* value are shown. Purple dots, hTau-Control. Blue dots, hTau-CRS. **(G,H)** Relative expression level of Angptl4 **(G)** and UCP2 **(H)** in the brain lysates from the hTau-Control and hTau-CRS mice. Data represent mean ± SEM (*n* = 6 for each group; **p < 0.01; unpaired *t*-test with Welch’s correction). **(I–K)** Representative immunoblots and quantification of PPAR-δ and Tau N368 protein expressions in the hippocampus of the hTau-Control and hTau-CRS mice. Data in **(I)** are representative of three independent experiments. Data in **(J,K)** represent mean ± SEM (*n* = 6 for each group; **p < 0.01; unpaired *t*-test with Welch’s correction). **(L)** Schematic of experiment procedures of 8-week-old WT or hTau mice, RS1: restraint stress for 1 day, RS14: restraint stress for 14 days, RS21: restraint stress for 21 days. **(M)** Schematic of experiment procedures of 8-week-old WT or hTau mice. **(N)** Representative immunoblots and quantification of Tau N368 protein expression in the hippocampus of the WT and hTau mice with no stress, resistant stress for 1 day (RS1), 14 days (RS14) and 21 days (RS21). Data in **(N)** are representative of three independent experiments. Data in **(O)** represent mean ± SEM (*n* = 6 for each group; **p < 0.01; one-way ANOVA and Bonferroni’s multiple comparison test). **(P,Q)** Representative immunoblots and quantification of Tau N368 protein expression in the hippocampus of the APP/PS1mice and 3xTg mice with no stress, resistant stress for 14 days (RS14). Data in **(P)** are representative of three independent experiments. Data in **(Q)** represent mean ± SEM (*n* = 6 for each group; **p < 0.01; one-way ANOVA and Bonferroni’s multiple comparison test).

Previous studies have proved that mitochondria dysfunction can be involved in both depressive symptoms and AD, while abnormal Tau could impair mitochondrial homeostasis (Xie et al., 2017; Fisar et al., 2019). Therefore, we hypothesized that mitochondrial dysfunction may be involved in the positive correlation of cognitive deficits and depressive-like behaviors in hTau mice exposed to CRS. As the hippocampus is a key anatomical structure for both memory decline and depression onset, the hippocampal mitochondrial function was measured in WT and hTau mice under stress (Figure 2A). We found that mitochondrial DNA expression, MMP and ATP production were significantly reduced only in the hippocampus of the hTau-CRS mice (Figures 2B–D). Notably, MMP were positively correlated with both discrimination index and sucrose preference in hTau mice (Figures 2E,F). These results indicate that mitochondrial dysfunction may play a key role in the co-morbidity of AD and depression.

PPAR-δ is pivotal in controlling mitochondrial biogenesis, and its suppression has been proved to trigger depression (He et al., 2021). Accordingly, we conducted qPCR to analyze the transactivation of PPAR-δ by measuring expression levels of PPAR-δ target genes, Angptl4 and UCP2. The results showed significant reductions in the expression levels of Angptl4 and UCP2 in the hTau-CRS mice, indicating the transactivation of PPAR-δ was strongly repressed in hTau mice exposed to CRS (Figures 2G,H). To understand why CRS repressed PPAR-δ transactivation in hTau mice, we measured the protein levels of PPAR-δ and Tau fragments in the hippocampus of hTau mice. The results showed that PPAR-δ levels were markedly decreased but Tau N368 levels were robustly increased in hTau-CRS mice (Figures 2I,J). To further investigate whether elevated Tau N368 fragment suppresses PPAR-δ through binding, we performed coimmunoprecipitation from the hippocampus of hTau mice. After immunoprecipitating Tau N368 with an antibody directed against it and immunoblotting with a PPAR-δ–specific antibody, we found that PPAR-δ interacted with Tau N368 fragment (Figures 2I,K). Next, we exposed 8-week-old WT and hTau mice to restraint stress (RS) for 1 day (RS1), or consecutively for 14 days (RS14) or 21 days (RS21), then quantified the protein levels of Tau N368 in hippocampus (Figure 2L). Hippocampal Tau N368 levels in both WT mice and hTau mice elevated with increased duration of RS, which is more obvious in hTau mice versus WT mice (Figures 2N,O). To further validate the RS-induced cleavage of Tau, we subjected 8-week-old APP/PS1 mice and 3xTg mice to control or RS14, followed measurement of Tau N368 levels (Figure 2M). The result showed Tau N368 was also elevated in the hippocampus of APP/PS1-RS14 and 3xTg-RS14 mice (Figures 2P,Q), with similarly suppressed transactivation of PPAR-δ (Supplementary Figures S1A–D), indicating Tau N368-PPAR-δ axis is ubiquitous in AD mouse models. Collectively, these findings indicate that Tau N368 fragments interact with PPAR-δ and suppress its mitochondrial protective function in CRS-AD models.

### Physical interaction of tau N368 with PPAR-δ triggers mitochondrial dysfunction

**Figure 3:**
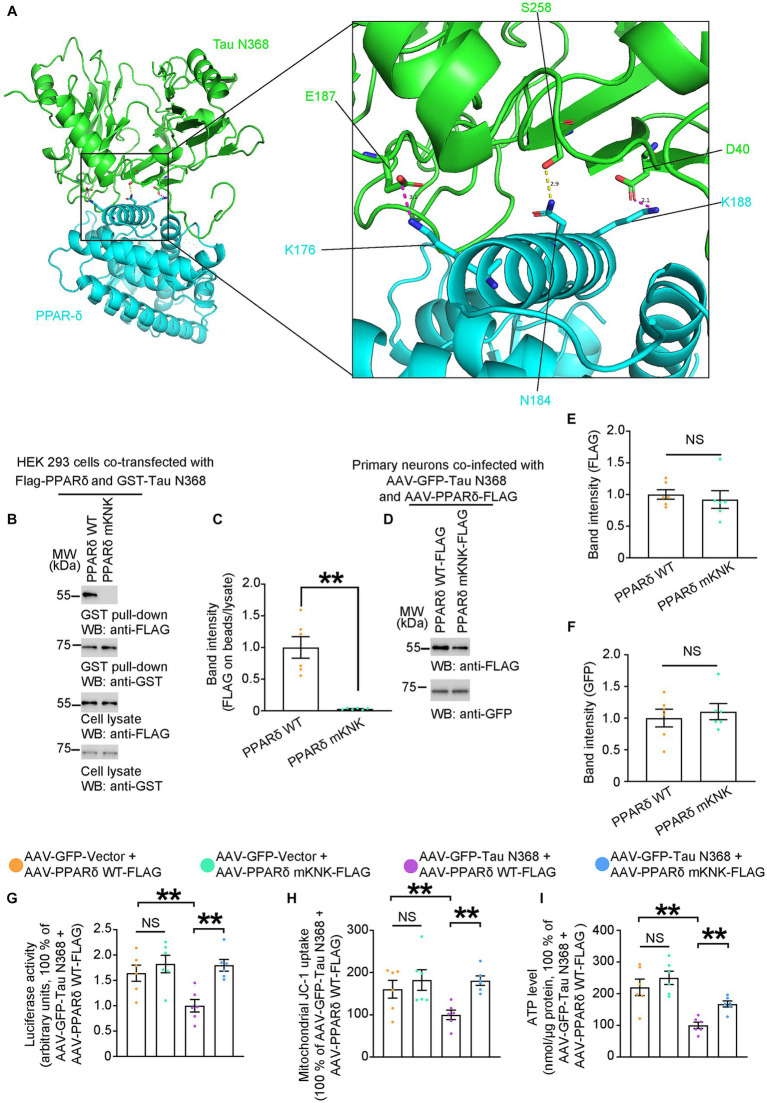
Physical interaction of Tau N368 with PPAR-δ triggers mitochondrial dysfunction. **(A)** Overall interaction and close-up view of between Tau N368 (green) and PPAR-δ (blue) are shown as ribbon models from the front view. The conserved salt bridges between E187-K176 and D40-K188 are colored purple, and the hydrogen bond between N184-S258 is colored yellow. **(B,C)** Representative immunoblots and quantification of FLAG and GST expressions in the GST pull-down and cell lysates from the HEK 293 cells co-transfected with Flag-PPARδ WT/mKNK and GST-Tau N368. Data in **(B)** are representative of three independent experiments. Data in **(C)** represent mean ± SEM (*n* = 6 for each group; **p < 0.01; unpaired *t*-test with Welch’s correction). **(D–F)** Representative immunoblots and quantification of FLAG and GFP expression in the cell lysates from primary neurons co-infected with AAV-GFP-Tau N368 and AAV-PPARδ WT/mKNK-FLAG. Data in **(D)** are representative of three independent experiments. Data in **(E,F)** represent mean ± SEM (*n* = 6 for each group; NS, not significant; unpaired *t*-test with Welch’s correction). **(G)** Primary neurons were co-infected AAV-GFP-Tau N368 or AAV-GFP-vector with AAV- PPARδ WT or AAV- PPARδ mKNK, then co-transfected with a 3 × PPRE-luciferase reporter with Renilla luciferase vector. Quantification of the luciferase activity is shown. Data are presented as the mean ± SEM (*n* = 6 for each group; **p < 0.01; unpaired *t*-test with Welch’s correction). **(H)** MMP, and **(I)** ATP levels in the primary neurons co-infected AAV-GFP-Tau N368 or AAV-GFP-vector with AAV- PPARδ WT or AAV- PPARδ mKNK. Data represent mean ± SEM (*n* = 6 for each group; **p < 0.01; unpaired *t*-test with Welch’s correction).

To address whether disruption of the interaction between PPAR-δ and Tau N368 could prevent neurotoxicity of Tau N368, we constructed expression constructs encoding PPAR-δ with deletions of different domains and co-transfected HEK 293 cells with GST-Vector or GST-Tau N368. After GST-pulldown, we found that the D region of the PPAR-δ was the main binding region for the Tau N368 (Supplementary Figure S2). Based on the above findings, we used computational protein–protein interaction prediction tool to identify and choose three possible binding sites in the D region of the PPAR-δ, K176, N184 and K188 (Figure 3A). To confirm the predicted binding sites, we constructed PPAR-δ mKNK (K176 to A176, N184 to A184, and K188 to A188) plasmid with the above three amino acids mutated to alanine (A). Subsequently, we performed GST-pulldown in HEK 293 cells co-transfected FLAG-PPAR-δ WT or FLAG-PPAR-δ mKNK with GST-Tau N368. The results showed that mKNK was sufficient to block the binding of PPAR-δ and Tau N368 (Figures 3B,C). To determine whether the binding of Tau N368 and PPAR-δ is necessary for mitochondrial dysfunction, primary cultured neurons were co-infected AAV-GFP-Tau N368 in combination with AAV-PPAR-δ WT-FLAG or AAV-PPAR-δ mKNK-FLAG. The expression levels of the viruses are shown in Figures 3D–F. Three days after infection, we co-transfected a 3 × PPRE-luciferase reporter with Renilla luciferase vector to primary neurons to evaluated the alterations in transcriptional activity of PPAR-δ. The mKNK mutation did not affect the basal transcriptional activity of PPAR-δ, while co-expressing Tau N368 significantly suppressed transactivation of PPAR-δ. As expected, the mKNK mutation significantly reversed the decreased transactivation of PPAR-δ induced by Tau N368 in primary neurons (Figure 3G). We next conducted measurement of mitochondrial function. The mutation of the binding sites in PPAR-δ significantly rescued Tau N368-mediated mitochondrial dysfunction in neurons (Figures 3H,I). These above results indicate that the physical interaction of Tau N368 with PPAR-δ is sufficient to induce neuronal mitochondrial impairment, which can be blunted by mutating the binding sites.

### Pharmacological inhibition of AEP downregulates tau N368 and restores depressive-like behaviors and cognitive deficits

**Figure 4:**
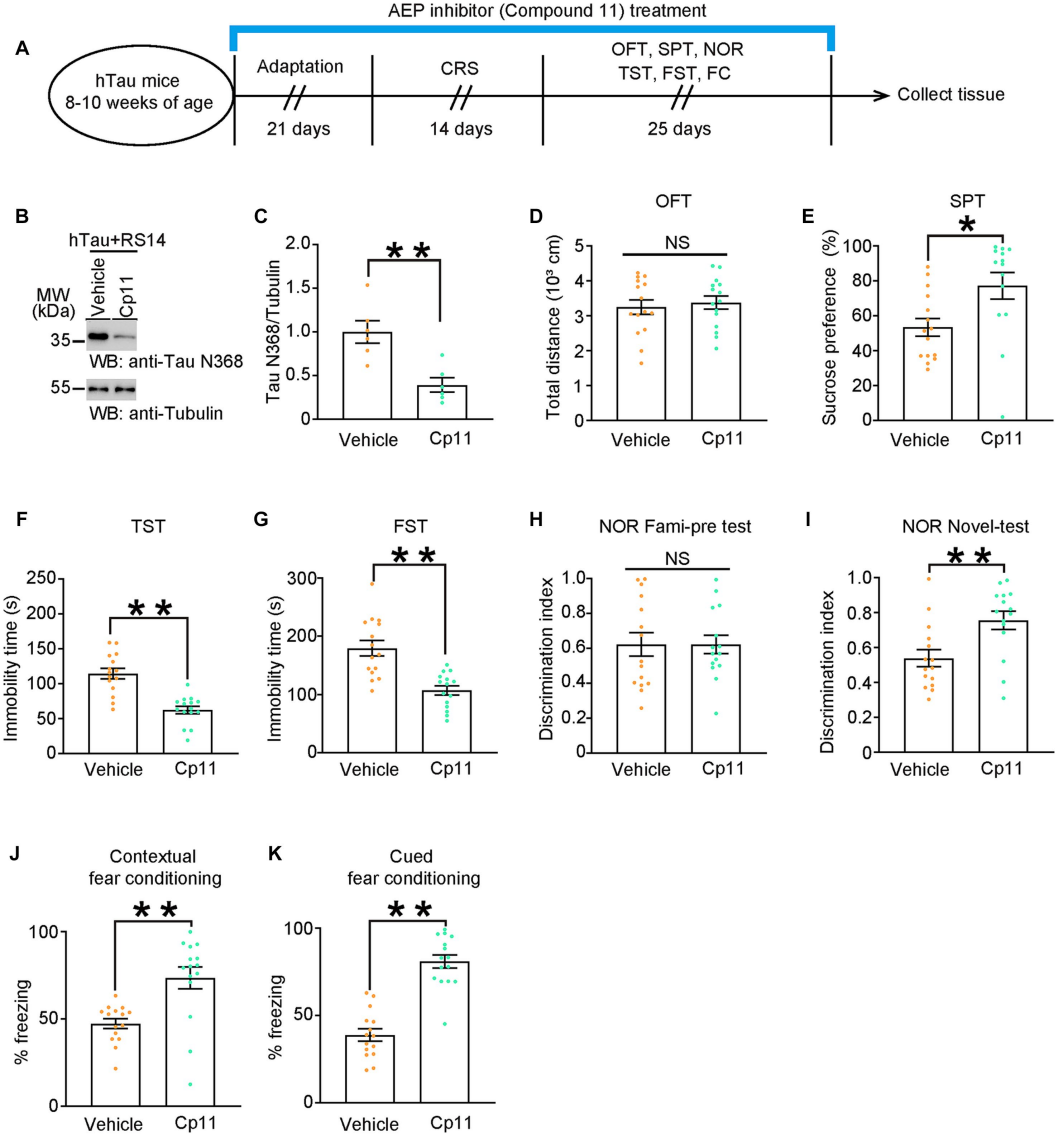
Pharmacological inhibition of AEP downregulates Tau N368 and restores depressive-like behaviors and cognitive deficits. **(A)** Schematic of the CRS and AEP inhibitor (Compound 11) or vehicle treatment, and behavioral tests process in hTau mice. **(B,C)** Representative immunoblots and quantification of Tau N368 expression in the hippocampus of hTau mice treated with Compound 11 or vehicle and 14 days of CRS. Data in **(B)** are representative of three independent experiments. Data in **(C)** represent mean ± SEM (*n* = 6 for each group; **p < 0.01; unpaired *t*-test with Welch’s correction). **(D)** Open-field test, **(E)** sucrose preference test, **(F)** tail suspension test, **(G)** forced swim test, **(H,I)** novel object recognition test, **(J)** contextual fear conditioning, **(K)** cued fear conditioning tests results. Experiments were performed on 8-10-week-old hTau mice with AEP inhibitor (Compound 11) or vehicle treatment after 14-day CRS. Results are presented as the mean ± SEM (*n* = 14–15 mice for each group; NS, not significant; *p < 0.05; **p < 0.01; unpaired *t*-test with Welch’s correction).

Based on previous research, an AEP inhibitor, compound 11, has oral bioactivity and shows therapeutic potential for treating AD (Zhang et al., 2017). It has been proved that compound 11 can reduce the production of Tau N368 fragment. Here, we treated hTau mice with compound 11 or vehicle once a day for consecutive 60 days. On day 22 of the treatment, mice were exposed to CRS and subsequent behavioral tests (Figure 4A). As shown by western blots, compound 11 significantly decreased the level of Tau N368 fragment in the hippocampus of hTau mice (Figures 4B,C). Compound 11 did not affect the locomotor activity of hTau mice (Figure 4D). Impressively, compound 11 successfully ameliorated the depressive-like behaviors and the cognitive deficits induced by CRS in hTau mice, as indicated by higher sucrose preference in the SPT, reduced immobility time in the TST and the FST, increased discrimination index in the NOR and less freezing time in the FCT (Figures 4E–K). These observations validate that reduction of Tau N368 fragment by compound 11 can restore cognitive deficiency and depressive-like behaviors induced by CRS in hTau mice.

### Anti-tau N368 treatment alleviates CRS-induced depressive-like behaviors and cognitive decline in AD mouse models

**Figure 5:**
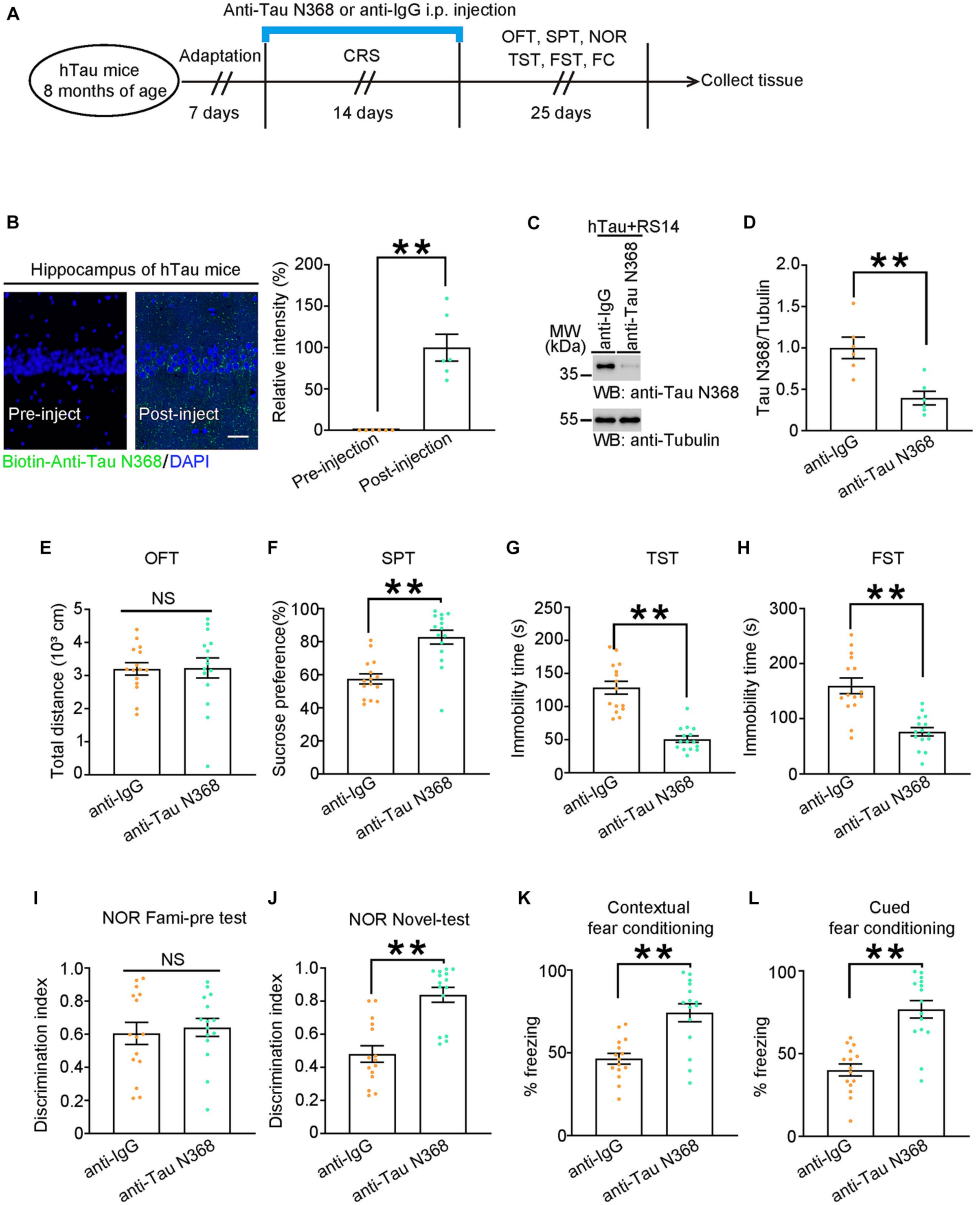
Anti-Tau N368 antibody treatment alleviates CRS-induced depressive-like behaviors and cognitive declines in AD mouse models. **(A)** Schematic of the CRS and Tau N368 antibody or IgG injection, and behavioral tests process in hTau mice. **(B)** Left: Immunofluorescence co-staining results of anti-Tau N368 antibody and DAPI in hippocampal slices from hTau mice before injection (left panel) or after injection (right panel) Scale bar: 50 μm. Right: quantification of relative immunofluorescent intensity of anti-Tau N368 antibody. **(C,D)** Representative immunoblots and quantification of Tau N368 expression in the hippocampus of the above mice. Data in **(C)** are representative of three independent experiments. Data in **(D)** represent mean ± SEM (*n* = 6 for each group; **p < 0.01; unpaired *t*-test with Welch’s correction). **(E)** Open-field test, **(F)** sucrose preference test, **(G)** tail suspension test, **(H)** forced swim test, **(I,J)** novel object recognition test, **(K)** contextual fear conditioning, **(L)** cued fear conditioning tests results. Experiments were performed on 8-month-old hTau mice with Tau N368 antibody or IgG injection during 14-day CRS. Results are presented as the mean ± SEM (*n* = 15 mice for each group; NS, not significant; **p < 0.01; unpaired *t*-test with Welch’s correction).

As AEP is involved in normal physiological processes and homeostasis, we administered intraperitoneal injections of anti-Tau N368 or anti-IgG antibody to hTau mice over a 14-day period of CRS and subsequently conducted behavioral tests (Figure 5A) to further validate the role of the Tau N368 fragment in the comorbidity of AD and depression. As shown by confocal images, biotinylated anti-Tau N368 antibody successfully penetrated the blood–brain barrier and entered neurons (Figure 5B). Western blots showed that anti-Tau N368 antibody successfully reduced the levels of Tau N368 fragment in the hippocampus of 8-month-old hTau mice (Figures 5C,D). Likewise, anti-Tau N368 or anti-IgG injection did not influence the mobility of hTau mice (Figure 5E). As expected, the injection of anti-Tau N368 antibody rescued memory declines and depressive-like behaviors induced by CRS in hTau mice aged 8 months (Figures 5F–L). Furthermore, we replicated the above experiments in 8-month-old 3xTg mice, another classic AD mouse model (Supplementary Figure S3A). Similarly, 3xTg male mice received Tau N368 antibody also showed unchanged mobility and their depressive-like behaviors and cognitive impairments were restored (Supplementary Figure S3B–I). These results suggest that directly reducing Tau N368 expression can alleviate cognitive dysfunction and emotional disorders resulted from CRS in AD mouse models.

### Pharmacological activation of PPAR-δ relieves CRS-induced depressive-like behaviors and cognitive decline in hTau mice

**Figure 6:**
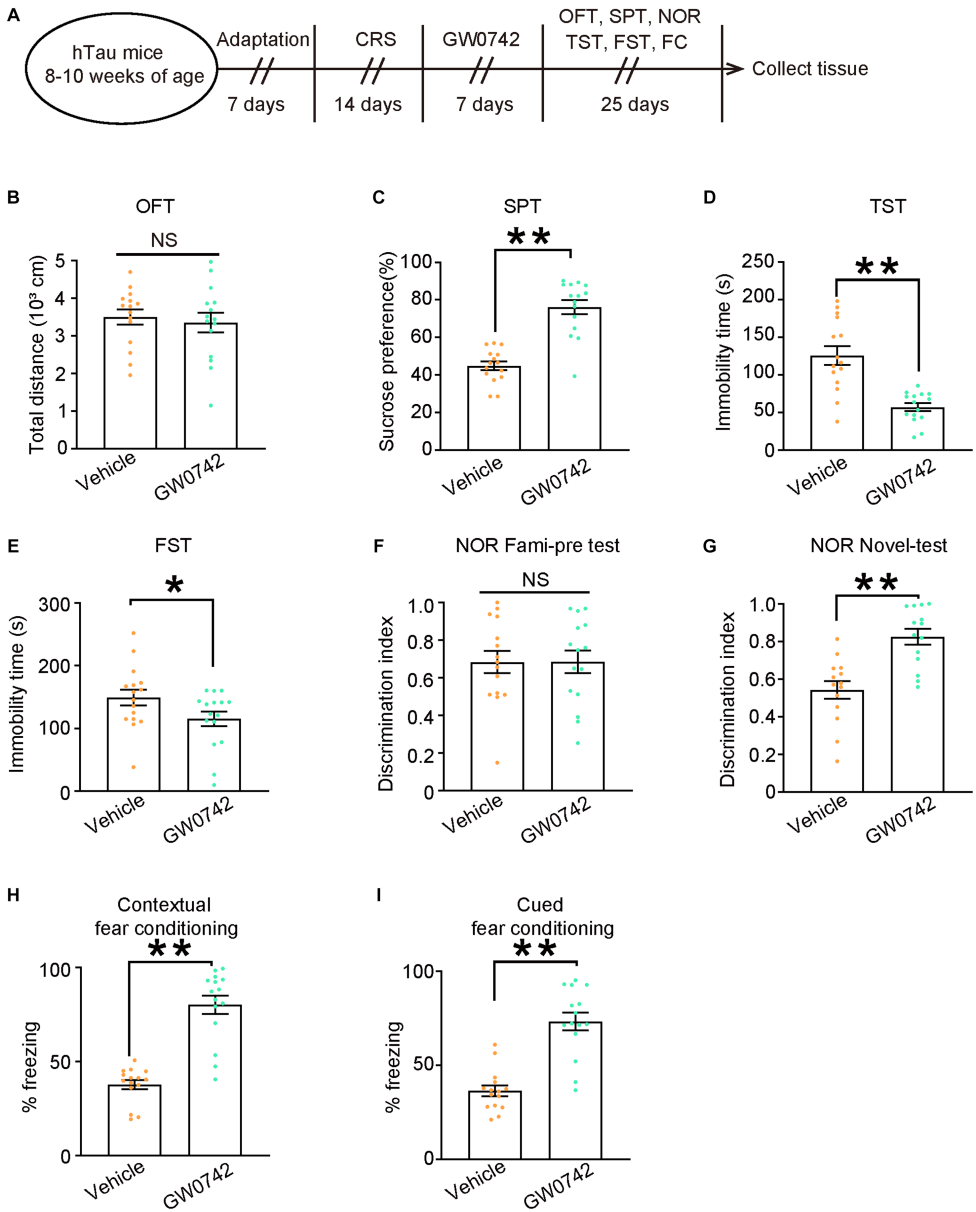
Pharmacological activation of PPAR-δ relieves CRS-induced depressive-like behaviors and cognitive decline in hTau mice. **(A)** Schematic of the CRS and GW0742 or vehicle treatment, and behavioral tests process in hTau mice. **(B)** Open-field test, **(C)** sucrose preference test, **(D)** tail suspension test, **(E)** forced swim test, **(F,G)** novel object recognition test, **(H)** contextual fear conditioning, **(I)** cued fear conditioning tests results. Experiments were performed on 8-10-week-old hTau mice with GW0742 or vehicle treatment following 14-day CRS. Results are presented as the mean ± SEM (*n* = 15–16 mice for each group; NS, not significant; **p < 0.01; unpaired *t*-test with Welch’s correction).

To address whether pharmacological activation of PPAR-δ attenuated behavioral abnormalities induced by CRS, we used selective PPAR-δ agonist GW0742 to treat hTau mice for 7 days following 14 days of CRS and then evaluated the behavioral outcomes (Figure 6A). Injection of GW0742 relieved depressive-like behaviors induced by CRS in hTau mice and did not impact their locomotor activity (Figures 6B–E). Simultaneously, impaired cognitive function induced by CRS was also alleviated by GW0742 in hTau mice (Figures 6F-I). Taken together, these results indicate that hypofunction of PPAR-δ is the key pathway in CRS-induced depressive-like behaviors and cognitive dysfunction.

## Discussion

Accumulating clinical studies have proved that individuals with AD exhibit neuropsychiatric symptoms, including anxiety, irritability, and depression (Mendez, 2021; Zong et al., 2022). Notably, depression is not only considered a prodromal stage of AD, but also have been linked to an increased risk of developing dementia in midlife or late life (Barnes et al., 2012). Depression would accelerate the course of dementia, while anti-dementia cholinesterase inhibitors could potentially have anti-depressant effects (Agüera-Ortiz et al., 2021). Moreover, 3-month-old APP/PSEN1-Tg mice *per se* showed depressive-like and anxiety-like behaviors, as well as memory impairments (Martín-Sánchez et al., 2021). These findings concluded that depression is highly associated with AD. In our study, we proposed a new perspective to explain the comorbidity of AD and depression that is Tau N368 fragment, elevated by CRS, directly binds PPAR-δ and represses its transactivation, leading to cognitive decline and depressive-like behaviors.

Neurons in the brain exhibit high energy demands and rely on mitochondrial ATP production. Mitochondria serve as the primary source of energy required for neurite outgrowth, and any deficiency in ATP results in impaired dendritic morphogenesis of neurons (Oruganty-Das et al., 2012). Emerging evidences have demonstrated that mitochondrial impairments may be associated in the pathophysiology of depression and neurodegenerative diseases (Allen et al., 2018; Pradeepkiran and Reddy, 2020).

PPAR-δ, a member of PPAR nuclear receptor family, is abundant in skeletal muscle and especially in brain (Luquet et al., 2003; Girroir et al., 2008). The main function of PPAR-δ is to regulate energy metabolism and promote mitochondrial biogenesis as a transcription factor (Christofides et al., 2021). Hypofunction of PPAR-δ leads to decreased mitochondrial DNA copy number, more oxidative damage (He et al., 2021). Accumulating studies showed that PPARs play a pivotal role in the pathophysiology of chronic stress-induced depressive phenotype. For instance, in chronic social defeat stress (CSDS)-induced mouse model, decreased hippocampal PPAR-α lead to reduction of BDNF expression, resulting in depressive-like behaviors(Song et al., 2018). Moreover, the transcriptional activity of PPAR-δ is suppressed in the mPFC of mice exposed to CSDS, thus contributing to mitochondrial dysfunction and depression-like phenotype. Activating PPAR-δ would rescue depressive-like behaviors in different chronic stress mouse models by restoring mitochondrial dysfunction (Tufano and Pinna, 2020; He et al., 2021). Furthermore, PPARs have been reported to participant in the progression of cognitive decline. Genetic knock-out of PPAR-δ impairs neuronal and synaptic structure, contributing to memory deficit in mice(Espinosa-Jiménez et al., 2022). Selective activation of PPAR-δ alleviates cognitive decline in mouse models of Alzheimer’s Disease(Malm et al., 2015) and type II diabetes mellitus(Abdel-Rahman et al., 2019). Additionally, a recent study revealed that suppression of PPAR-δ transactivation contributes to the onset and progression of Huntington’s disease (HD) (Dickey et al., 2016). In the present study, we found that CRS would elevate Tau N368 fragment derived from AEP-mediated cleavage, which subsequently repressed PPAR-δ by physically interaction, leading to mitochondrial dysfunction. Following blockage of the binding between Tau N368 and PPAR-δ, the mitochondrial impairment was successfully mitigated in the primary neurons. Collectively, our study has identified Tau N368-PPAR-δ axis as an upstream regulatory mechanism of mitochondrial dysfunction, which contributes to the CRS-induced depressive-like behaviors and cognitive deficits.

Our previous studies have reported that Tau N368 fragment is produced by AEP-mediated cleavage and plays an important role in neurodegenerative diseases like AD and Parkinson’s disease (PD) (Zhang et al., 2014; Xiang et al., 2019; Ahn et al., 2020; Kang et al., 2020). Additionally, compound 11, an AEP inhibitor, can diminish the production of Tau N368 and ameliorate cognitive impairment in mouse models of AD (Kang et al., 2020). However, there is a lack of research about the role of Tau N368 in emotional disorders. In our current study, we reported that Tau N368 was sufficient to induce depressive-like behaviors in AD mouse models. Moreover, the pharmacological inhibition of AEP using compound 11, the clearance of Tau N368 via antibody administration, and the application of a PPAR-δ agonist all demonstrated significant efficacy in alleviating the depressive-like behaviors and cognitive impairments induced by CRS in mice. The above results have the potential to shed light on the treatment of co-morbidity involving AD and depression.

Our current study identified a novel post-translational modification of Tau disturbs PPAR-δ-mediated mitochondrial homeostasis. A previous study validated Tau hyperphosphorylation in the cortex of PPARβ/δ-null mice (Barroso et al., 2013). Notably, PPARβ/δ-null mice shows intrinsic impairment in cognition, accompanied with elevated pro-inflammatory cytokines in cortex (Barroso et al., 2013). We have reported that AEP is transcriptionally upregulated by an inflammatory cytokine-activated transcription factor, CCAAT/enhancer binding protein β (C/EBPβ)(Wang et al., 2018). Therefore, it is possible that chronic stress-reduced PPAR-δ may trigger neuroinflammation, which subsequently activating C/EBPβ-AEP axis and eventually increasing Tau N368 levels. This speculation would be further investigated in the future.

Collectively, our work highlights the critical role of Tau N368-PPAR-δ axis in hippocampal neurons in modulating mitochondrial dysfunctions and causing depressive-like behaviors and cognitive declines. These results provide valuable insights into the underlying mechanisms of the depressive symptoms in AD, and offer a novel target for further refining therapeutic approaches to depressive disorders and AD.

## Data availability statement

The raw data supporting the conclusions of this article will be made available by the authors, without undue reservation.

## Ethics statement

The animal study was approved by Laboratory Animal Welfare Ethical Committee of Renmin Hospital of Wuhan University. The study was conducted in accordance with the local legislation and institutional requirements.

## Author contributions

YW: Conceptualization, Data curation, Formal analysis, Funding acquisition, Methodology, Software, Supervision, Validation, Visualization, Writing – original draft, Writing – review & editing. JianW: Conceptualization, Data curation, Formal analysis, Methodology, Project administration, Software, Visualization, Writing – review & editing, Writing – original draft. HC: Conceptualization, Data curation, Investigation, Methodology, Supervision, Writing – review & editing, Writing – original draft. XL: Data curation, Formal analysis, Investigation, Methodology, Project administration, Resources, Supervision, Visualization, Writing – review & editing. RX: Methodology, Software, Writing – review & editing. FG: Data curation, Project administration, Writing – review & editing. HY: Data curation, Investigation, Project administration, Validation, Writing – review & editing. FL: Data curation, Investigation, Methodology, Project administration, Validation, Writing – review & editing. DQ: Investigation, Writing – review & editing. JiaW: Conceptualization, Project administration, Validation, Writing – review & editing. YS: Formal analysis, Investigation, Investigation, Writing – review & editing. YL: Formal analysis, Validation, Writing – review & editing. SL: Data curation, Project administration, Validation, Visualization, Writing – review & editing. XZ: Formal analysis, Visualization, Writing – review & editing. SD: Data curation, Writing – review & editing. YH: Formal analysis, Writing – review & editing. LH: Data curation, Investigation, Project administration, Writing – review & editing. X-YG: Conceptualization, Methodology, Project administration, Visualization, Writing – original draft, Writing – review & editing. ZL: Conceptualization, Methodology, Project administration, Writing – original draft, Writing – review & editing. JL: Data curation, Investigation, Methodology, Project administration, Writing – original draft, Writing – review & editing. Z-HW: Funding acquisition, Investigation, Methodology, Project administration, Validation, Writing – original draft, Writing – review & editing.
