# Supplementary figures and images for: A tau fragment links depressive-like behaviors and cognitive declines in Alzheimer’s disease mouse models through attenuating mitochondrial function
Source: Front Aging Neurosci. 2023 Dec 6;15:1293164. doi: 10.3389/fnagi.2023.1293164 (PMC10734641; doi:10.3389/fnagi.2023.1293164)

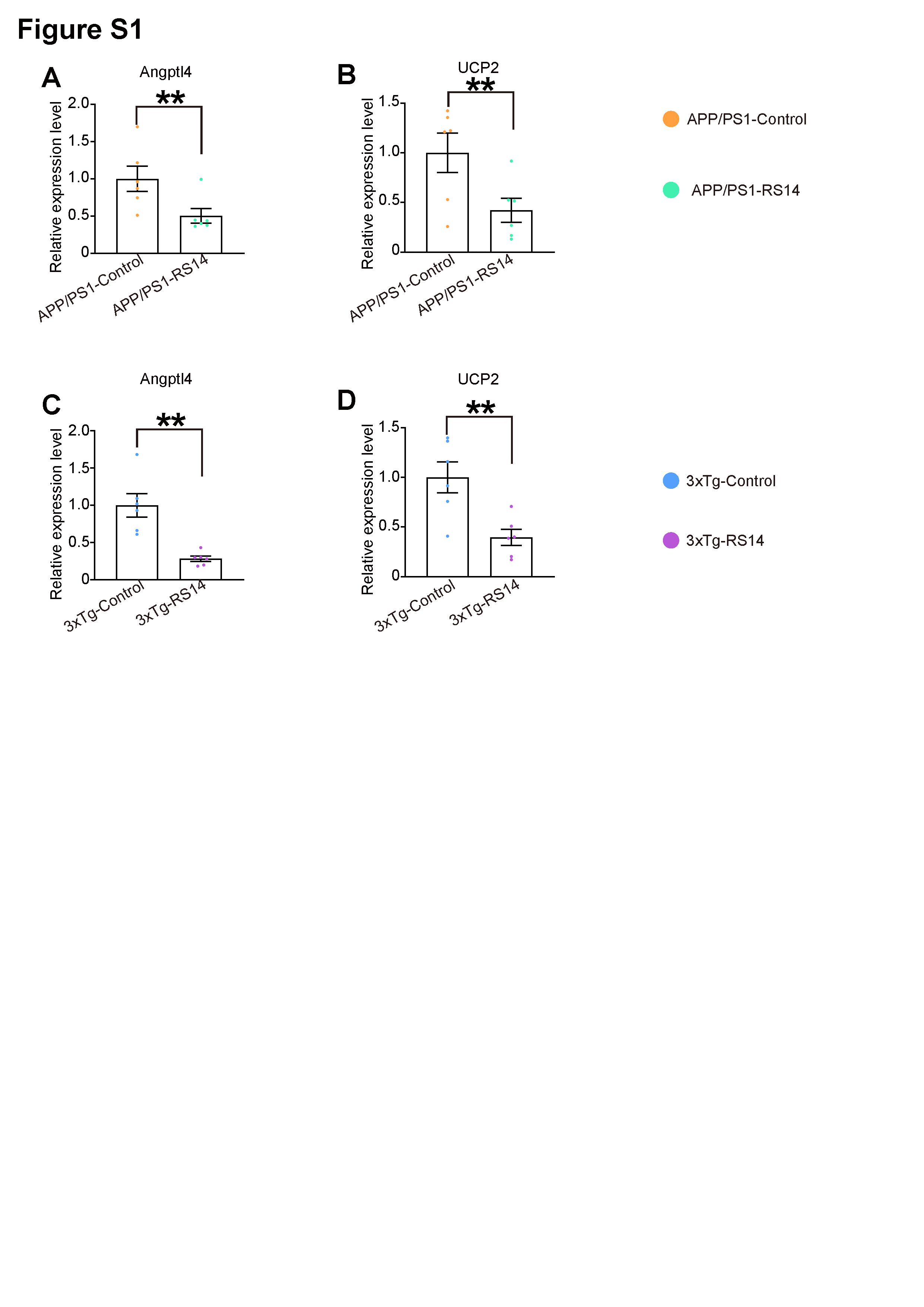

Supplement: Supplementary file 1 [file Image_1.tiff]

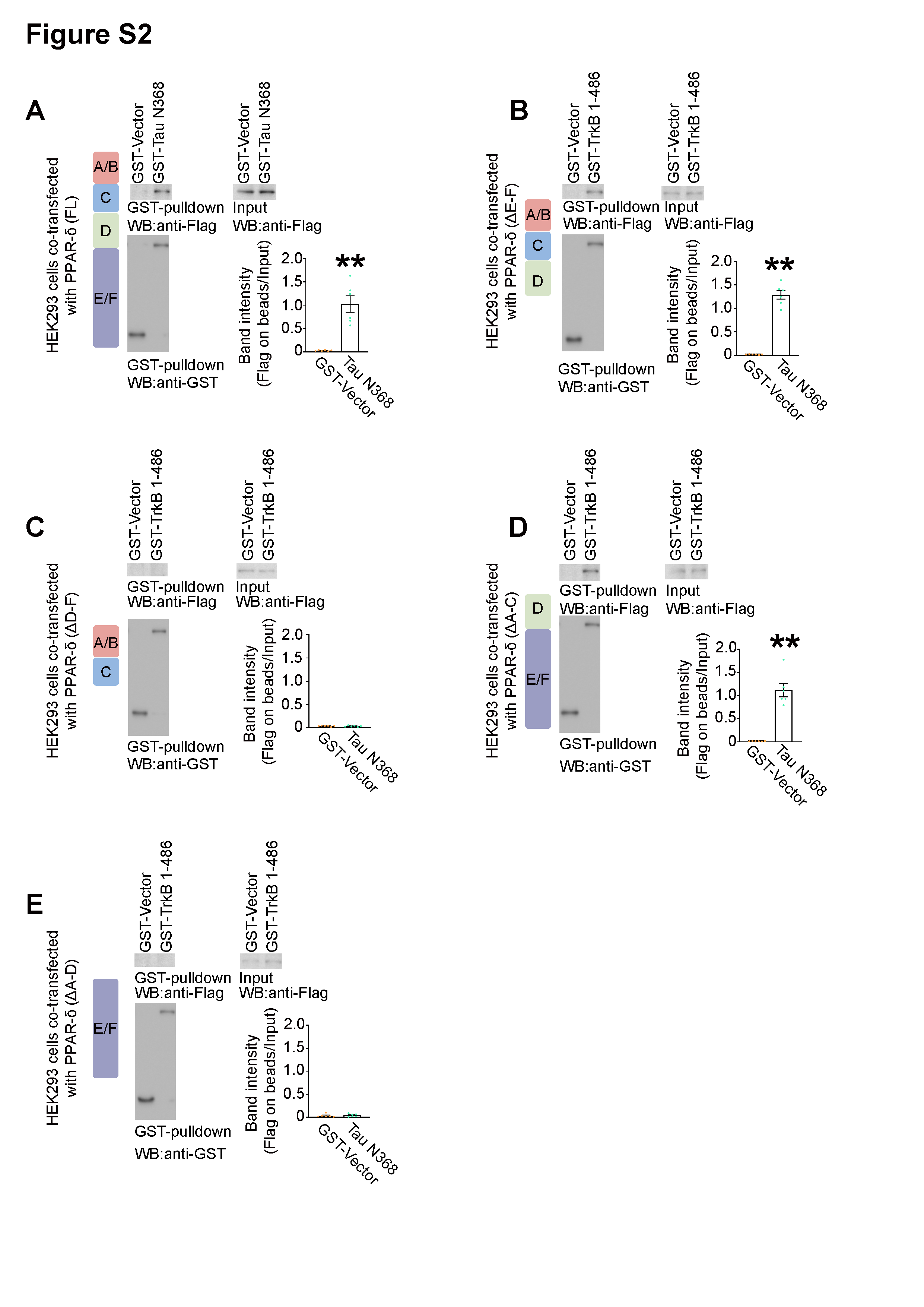

Supplement: Supplementary file 2 [file Image_2.tiff]

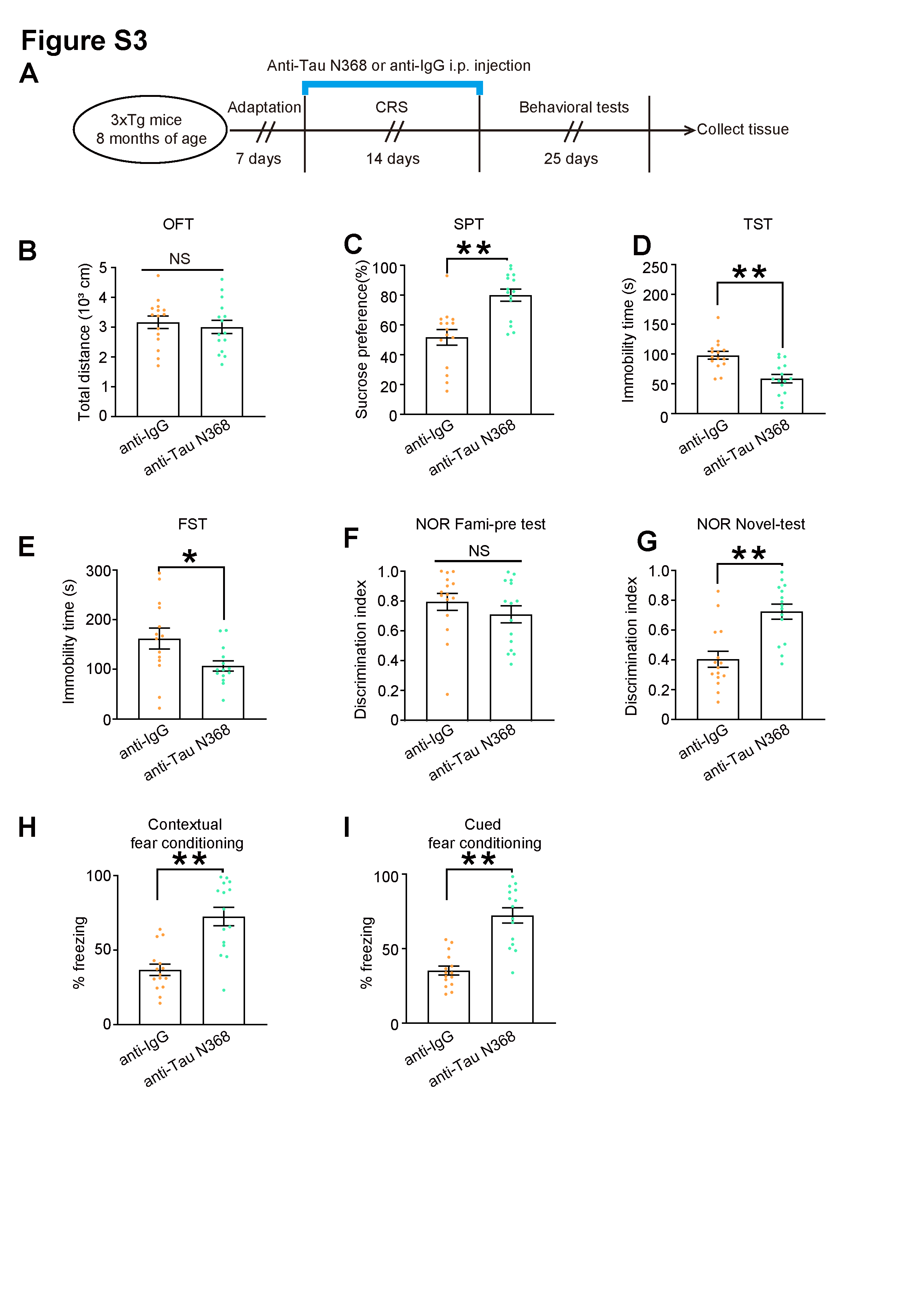

Supplement: Supplementary file 3 [file Image_3.tiff]
